# Implementation of Traffic Light System on food sold at Brockfield House Medium secure unit to help improve healthy food options

**DOI:** 10.1192/j.eurpsy.2022.1544

**Published:** 2022-09-01

**Authors:** N. Agunwamba, D. Ho, M. Duffy

**Affiliations:** Essex Partnership University Foundation Trust, Forensic Psychiatry, Runwell Wickford Essex, United Kingdom

**Keywords:** Secure Services, forensic psychiatry, lifestyle choices, obesity

## Abstract

**Introduction:**

Public Health England published a report in 2017 on Obesity in Secure Mental Health units. A key finding was that not only is obesity and overweight more prevalenr in the population detained within mental health secure units (reported rates of up to 80%) than in the general population (around 60%), patients appear to be more at risk of weight gain when they are detained.

**Objectives:**

1.To implement a traffic light system on food and confectionaries sold at the shop at a Medium secure hospital. 2. Provide healthier food options at the shop by using traffic light system as a visual aid 3. To achieve weight reduction and promote healthy life style choices in patients admitted to our medium secure Forensic unit

**Methods:**

1.Buying a new till system which is able to quantify what type of food is sold 2.Labelling food sold using traffic light system 3. Calculate the types of food sold following a three- month period after implementation.

**Results:**

/Intended Outcome Traffic light system provides a visual aid to patients in choosing healthier food Patients in our medium secure unit achieve a reduction in their weight Traffic light system can be replicated/ adopted by other secure hospitals

**Conclusions:**

The purpose of this research is to implement a traffic light system on food sold at a shop in our medium secure unit and that this will help improve food choices in the unit.

**Disclosure:**

No significant relationships.

